# Evidence of Chinese herbal fumigation for knee osteoarthritis: A protocol for systematic review and meta-analysis

**DOI:** 10.1097/MD.0000000000024532

**Published:** 2021-02-12

**Authors:** Liang Ou, Yingfu Meng, Zehua Chen, Tiantian Zhang, Dezhong Kong, Daoqing Xu, Weichen Huang

**Affiliations:** a The Second Affiliated Hospital of Guizhou University of Chinese Medicine, Guiyang; b The Fifth Clinical Medical School, Guangzhou University of Chinese Medicine, Guangzhou; c The Hunan University of Chinese Medicine, Changsha, China.

**Keywords:** fumigation, osteoarthritis, protocol, systematic review, traditional chinese medicine

## Abstract

**Background:**

Knee osteoarthritis (KOA) is the most common cause of musculoskeletal pain and disability worldwide. Chinese herbal fumigation, an external therapy, is commonly used for the treatment of KOA, while there is no systematic review or meta-analysis designed to evaluate the effects of Chinese herbal fumigation on KOA.

**Methods:**

Seven databases including Cochrane Central Register of Controlled Trials, EMBASE, PubMed, China National Knowledge Infrastructure, Wanfang data, VIP, and Chinese Biomedical Literature Database will be searched up to October 31, 2020. Data that meet the inclusion criteria will be extracted and analyzed by using RevMan V.5.3 software. Two reviewers will assess quality of the included studies based on the Cochrane Collaboration risk of bias tool. The funnel plot and Begg test will be used to evaluate publication bias. And Grading of Recommendations Assessment, Development and Evaluation will be employed to assess the quality of evidence.

**Results:**

This study will provide high-quality evidence for Chinese herbal fumigation for the treatment of KOA in terms of effectiveness and safety.

**Conclusion:**

This systematic review will provide evidence to help us confirm the clinical efficacy of Chinese herbal fumigation in the treatment of KOA.

## 1 Introduction

Osteoarthritis (OA), as the most common musculoskeletal progressive disorder, leads to pain, loss of function, and decreased quality of life in elderly people.^[1]^ Conventionally, it affects the predominant weight-bearing joints, such as the hips and knees, of which knee osteoarthritis (KOA) is the most common type.^[2]^ It is estimated that OA affects about 1/3 people over the age of 60, with a disability rate as high as 53%.^[3]^ An epidemiological study showed that 8.9% of the adult people experienced clinically significant-KOA.^[4]^ On the basis of a nationwide population-based study in China, the prevalence of KOA in women and men was 42.8% and 21.5% respectively, of which the symptomatic knee osteoarthritis was 8.1%.^[5]^ Being a chronically joint disease, KOA is mainly characterized by degeneration of the articular cartilage, thickening of the subchondral bone, osteophyte formation, variable degrees of synovial inflammation, and degeneration of the entire joint structure, including the synovium, periarticular ligament, and meniscus (in the knee).^[6]^ Until now, the pathogenesis of KOA is not elucidated; however, it is understood to be a complex interaction of local and systemic factors.^[7]^ Major risk factors for KOA include age, gender, obesity, overuse, joint injury, genetic predisposition, and mechanical factors.^[8]^ At present, there is no radical treatment for KOA. Therefore, the main objectives in the management of KOA still focus on alleviating pain and delaying progression into end-stage KOA, which needs for knee arthroplasty to decreases pain and improves function. Although knee arthroplasty is a very effective treatment approach for advanced osteoarthritis of the knee, some patients achieve poor results after surgery or the implant fails, such as infection, prosthesis loosening and so on.^[9]^ Undoubtedly, non-surgical treatment, comprising non-pharmacological and pharmacological treatment, serves as core first-line treatment for KOA patients. Standard pharmacological therapies always choose oral or topical non-steroidal anti-inflammatory drugs, hyaluronic acid injections, or intra-articular corticosteroids. However, these drugs relate to some inevitable adverse effects, such as hepatorenal toxicity, gastrointestinal reactions, and adverse events with the risk of cardiovascular.^[10,11]^ Thus, it is necessary to develop other safer and more effective treatments for preventing further progression or delaying the onset of end-stage arthritis of the knee. Traditional Chinese medicine (TCM), as an important part of complementary and alternative medicine, is prevalent in Asia and has been proven to exert therapeutic effects on KOA. A recent study suggested that TCM was associated with a reduced need for total knee replacement in patients with KOA, with increased benefits from longer durations of TCM use.^[12]^

Chinese herbal fumigation is an external therapy, including fumigation and washing, which fumigates and washes the skin or affected area while it is hot after decoction of traditional Chinese medicine prescription consisting of specific medicinal ingredients. Chinese herbal fumigation therapy has analgesic and anti-inflammatory effects, as well as activate blood circulation which will attribute to relieve symptoms of pain.^[13]^ Although Chinese herbal fumigation treatment has been commonly applied to treat osteoarthritis for thousands of years in China, the evidence for quantitative analysis to examine treatment effects remains lacking. To our knowledge, few researches have systematically assessed all previously published controlled trials of Chinese herbal fumigation treatment for KOA. Therefore, the purpose of this study is to precisely evaluate current evidence related to the effectiveness and safety of Chinese herbal fumigation on KOA, which will provide a reference for the treatment of KOA in clinical practice.

## 2 Methods

### 2.1 Study registration

We will perform this meta-analysis according to the Preferred Reporting Items for Systematic Reviews and Meta-analyses.^[14]^ The protocol of this review has been registered with the Open Science Framework (OSF, https://osf.io/pnw6r), and the registration DOI of this study is 10.17605/OSF.IO/PNW6R.

### 2.2 Inclusion criteria for study selection

#### 2.2.1 Study design

All the published articles of clinical randomized controlled trials related to the efficacy of Chinese herbal fumigation treatment on KOA will be considered for inclusion. However, the following articles will be excluded: observational studies, animal experiments, reviews, full-text unavailable studies, and duplicate publications.

#### 2.2.2 Participants

Participants who meet the diagnosis of KOA, regardless of the early-stage or end-stage, will be included in this review. People suffering from secondary KOA or other non-suppurative arthritis, such as traumatic arthritis, rheumatoid arthritis, and allergic arthritis, will be excluded.

#### 2.2.3 Interventions

In randomized controlled trials, the group including Chinese herbal fumigation is defined as the experimental group. The control group included basic treatments, blank control, or placebo. We will include the 3 comparisons between experimental and control group: Chinese herbal fumigation with basic treatments versus basic treatments; Chinese herbal fumigation versus basic treatments; Chinese herbal fumigation versus no intervention. Any interventions comprised of surgery or other complementary and alternative medical treatments, such as acupuncture and Chinese medicine, will be excluded.

#### 2.2.4 Outcome measures

In this review, the primary outcomes will include joint pain (scored by Visual Analog Scale or Numerical Rating Scale) and function (evaluated by Western Ontario and McMaster Universities Arthritis Index, Lysholm, or Bisrotl scores.) after treatment. We will regard quality of life (using SF-36), effective rate, adverse events and related biomarkers as the secondary outcomes.

### 2.3 Search methods for the identification of studies

#### 2.3.1 Electronic searches

Seven databases including Cochrane Central Register of Controlled Trials, EMBASE, PubMed, China National Knowledge Infrastructure, Wanfang data, Chinese Scientific Journal Database (VIP), and Chinese Biomedical Literature Database will be searched up to October 31, 2020. The following search terms: knee osteoarthritis, osteoarthritis, arthritis, fumigation, bath, and wash, will be applied to retrieval without restrictions.

#### 2.3.2 Other searches

In order to supplement the insufficient of the database searches, we will browse related academic websites including Google Scholar and Baidu Academic. Meanwhile, the reference lists of eligible articles and gray literature will be performed manually.

### 2.4 Study selection and data extraction

**Figure 1:**
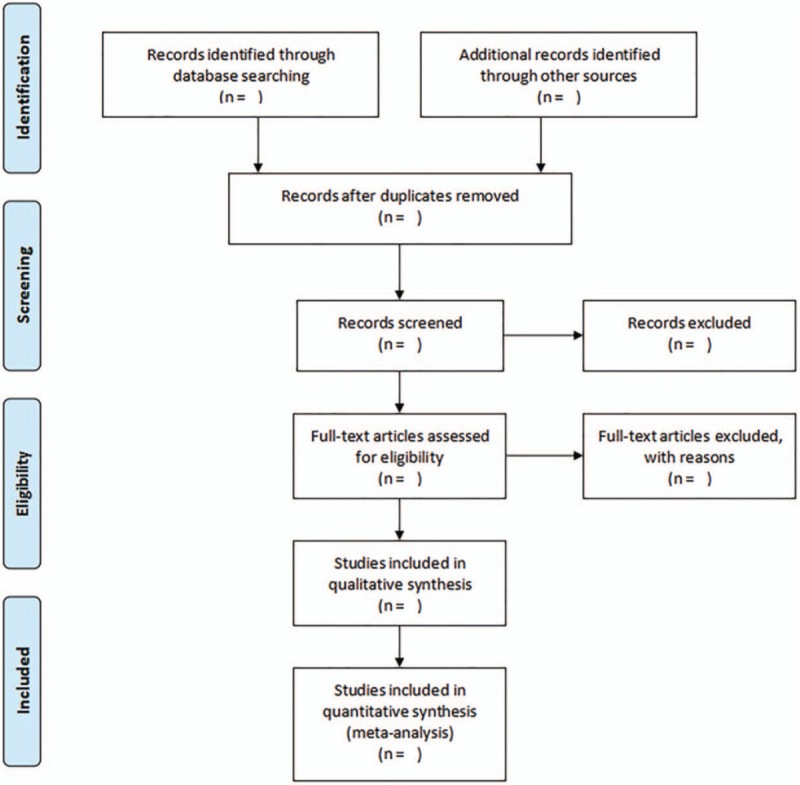
PRISMA flow diagram of study selection process.

All the articles retrieved will be imported into the Endnote X9 software. First, reduplicative literatures will be removed. Second, we will delete those studies apparent inconformity with inclusion criteria by reading the titles and abstracts. Subsequently, the eligible literatures will be collected according to the inclusion and exclusion criteria after reading the full text of the remaining literatures carefully. Finally, we will extract the main information of the included studies, including authors’ name, year of publication, sample size, gender and age of participants, study design, diagnosis criteria, interventions of treatment and control group, follow-up, and outcome measures. The above-mentioned work will be executed independently by 2 researchers (L Ou and YF Meng). If there is any disagreement, it will be resolved in consulting with the third researcher (WC Huang). The selection procedure of this review will be summarized by using a PRISMA flow diagram (Fig. 1).

### 2.5 Quality assessment

Two reviewers (L Ou and YF Meng) will make use of the Cochrane collaboration's tool for assessing risk of bias to assess the methodological quality of the including literature respectively. Controversy, if any, will be decided by discussion with the third reviewer (WC Huang). The assessment tool consists of 7 aspects: random sequence generation, allocation concealment, blinding for participants and researchers, blinding for outcome assessment, integrity of outcome data, selective report and other bias. Meanwhile, each aspect is divided into 3 levels: ‘low,’ ‘unclear,’ and ‘high’^[15]^

### 2.6 Data synthesis and analysis

Meta-analysis will be performed by using the RevMan 5.3 software provided by Cochrane Collaboration. The continuous variables will be pooled by the standardized mean difference (SMD) with 95% confidence interval (CI), while for binary variables, the odds risk (OR) with 95% CI will be pooled. Heterogeneity will be determined by the Cochran *Q*-test and *I*^*2*^ value. If *I*^*2*^ <  = 50%, it is considered that there is no statistical heterogeneous. Then we will apply the fixed effects models to conducting the meta-analysis. If *I*^*2*^ > 50%, it means there is substantially heterogeneous. However, the random effects models will be employed for the meta-analysis. A subgroup analysis or sensitivity analysis, if necessary, will be performed to explore the source of heterogeneity. If fails, only descriptive analysis will be used. A value of *P* < .05 is considered to be statistically significant.

### 2.7 Assessment of reporting bias

Publication bias will be analyzed by the funnel plot and the Begg test.^[16]^

### 2.8 Grading the quality of evidence

The software of GRADE profiler 3.6 will be recommended to assess the quality of evidence for all outcomes, which will be divided into 4 levels: high, medium, low, and very low.^[17]^

### 2.9 Dealing with missing data

We will contact the author of the included articles by email or telephone, when all the necessary data cannot be obtained from the literature. If fails, then the study will be eliminated, and the potential impact of the missing data on the results will be explained further in the discussion.

### 2.10 Ethics

Ethics approval is not necessary for this study because we will only analyze published literatures.

## 3 Discussion

Knee osteoarthritis is the most common form of joint disease in humans. Pain and dysfunction caused by KOA severely decreases the quality of their life.^[18]^ Until now, the etiology of KOA is remains unclear and there is no radical cure for KOA. With continuous exploration for the treatment of KOA, an increasing number of non-surgery therapies has been proposed. Although pharmacological approaches including non-steroidal anti-inflammatory drugs, opioid analgesics and sodium hyaluronate are widely used in clinical practice for the treatment of KOA, the clinical efficacy achieved by these medicines is insufficient.^[2,19]^ Moreover, doctors and patients concern constantly about the potential adverse effects of these drugs. Chinese herbal fumigation is proved to have therapeutical effects with analgesic, anti-inflammatory, and activate blood circulation. However, the efficacy and safety of Chinese herbal fumigation on KOA is still lack of evidence. There has been no systematic review evaluating its therapeutical effects. In the discussion section of this paper, we will include the following aspects:

(1) summary of main findings;
(2) comparison with other studies;
(3) interpretation of the results;
(4) strength and limitations;
(5) conclusion.

Therefore, we hope this study will provide a summary of the current state of evidence regarding the safety and efficacy of Chinese herbal fumigation in treating knee osteoarthritis.

## Author contributions

**Conceptualization:** Liang Ou, Yingfu Meng.

**Data curation:** Dezhong Kong, Daoqing Xu.

**Formal analysis:** Zehua Chen, Tiantian Zhang.

**Methodology:** Weichen Huang.

**Project administration:** Weichen Huang.

**Resources:** Weichen Huang.

**Software:** Dezhong Kong, Daoqing Xu.

**Supervision:** Weichen Huang.

**Writing – original draft:** Liang Ou.

**Writing – review & editing:** Yingfu Meng, Weichen Huang.

## Abbreviations

Abbreviation: KOA = knee osteoarthritis.
